# Use and overuse of triptans in Austria – a survey based on nationwide healthcare claims data

**DOI:** 10.1186/s10194-018-0864-0

**Published:** 2018-05-18

**Authors:** Karin Zebenholzer, Walter Gall, Christian Wöber

**Affiliations:** 1 Department of Neurology, Medical University of Vienna, Waehringer Guertel 18-20, 1090 Vienna, Austria; 2 Center for Medical Statistics, Informatics and Intelligent Systems, Institute of Medical Information Management, Medical University of Vienna, Waehringer Guertel 18-20, 1090 Vienna, Austria

**Keywords:** Triptan, Migraine, Medication overuse headache, Triptan overuse

## Abstract

**Background:**

To evaluate triptan use and overuse as well as prescription patterns in Austria based on a nationwide healthcare database because data on triptan use and overuse in Austria is missing.

**Methods:**

We included all persons insured with one of 19 Austrian social security institutions in 2007. Inclusion criteria comprised an age of 18–99 years, known sex, and receipt of insurance benefits. We defined triptan use as ≥1 package of a triptan dispensed in 2007 and triptan overuse as ≥30 defined daily doses dispensed in at least one quarter.

**Results:**

Out of 8.295 million inhabitants in Austria, 7,426,412 persons (89.5%) were insured with a social insurance carrier and 5,918,487 persons of those insured (79.7%) fulfilled the inclusion criteria. Among the latter 33,062 persons (0,56%) were triptan users and 1970 (0.033%) were triptan overusers. The estimated proportion of persons with migraine using a triptan was less than 6%. Among users 5.9% were overusers of whom 55% overused triptans in ≥2 quarters of 2007. The median number of days of sick-leave was higher in triptan users than in non-users: due to any reason of sick-leave 12 vs. 10, *p* < 0.001, due to migraine 3 vs. 2, *p* < 0.001. The proportion of hospital admissions did not differ between triptan users and non-users.

**Conclusion:**

The rate of triptan use is low in Austria but triptan users are at risk for triptan overuse. In triptan users more days of sick-leave and the same proportion of hospital admissions as in the older non-users suggest poorer health.

## Background

Migraine shows one-year prevalence rates of 10–18% and is associated with a substantial burden [1]. In the Global Burden of Disease Study, migraine was the second most common disorder after tension-type headache and the second largest contributor to disability-adjusted life years (DALYs) after stroke [2]. Annual direct and indirect costs per person caused by migraine were estimated at 1222 Euros rising to 3700 Euros in chronic migraine in 2008 and 2009 [3, 4]. These costs are largely due to sick leave and presenteeism [3].

The management of migraine comprises treatment of acute attacks and prophylaxis. Acute migraine attacks are treated with analgesics, nonsteroidal anti-inflammatory drugs (NSAIDS), or triptans [5]. Triptans are selective serotonin agonists acting at 5-HT1B/1D receptors. Non-pharmacological and pharmacological prophylaxis is required in patients with three or more migraine attacks per month [5]. It is particularly important in patients with frequent use of acute migraine medication to prevent medication overuse headache which affects approximately 1% of the general population [6]. In specialized headache centres in Austria more than 40% of the patients with chronic headache had (probable) medication overuse headache [7].

Furthermore, the management of migraine requires considering comorbidities such as depression and anxiety disorders [8]. Their prevalence in patients with migraine was estimated two to 10 times that of the general population with higher rates in chronic migraine [8]. Depression and anxiety have a negative impact on migraine [8, 9], and the use of antidepressants was reported in 4–49% of the patients with migraine [10–12]. Persons using triptans together with serotonergic antidepressants or other serotonergic drugs may be at risk of a serotonin syndrome [13].

In previous pharmacoepidemiological studies, the proportion of triptan users was low whereas triptan overuse was not uncommon: In recent studies from the Netherlands, Italy, and France the proportion of triptan users ranged from 0.7–2.3% of the examined populations of whom 3.3–10% were overusers [10, 14, 15]. Older studies reported triptan use in 0.55–1.4% of the populations and overuse in 0.9–14.3% of the users [16–20]. However, these older studies were restricted to certain regions or certain insurance requirements or were based on pharmacies’ datasets.

In Austria, data on the use and overuse of triptans is missing. A nationwide research database allowed to overcome this lack of information. The General Approach for Patient-oriented outpatient-based Diagnosis Related Groups (GAP-DRG) database of the Main Association of Austrian Social Security Institutions (Hauptverband der Österreichischen Sozialversicherungsträger) provides anonymous data on dispensed drugs, sex, age, and other details for particular years. The database covers the vast majority of Austria’s population as each inhabitant, with minor exceptions, has to be insured with a social security institution. The responsible carrier is set by law and cannot be chosen freely. We used the GAP-DRG database to assess the use and overuse of triptans and to explore differences between the nine provinces as well as between urban and rural regions. In addition, we analysed the prescription of migraine prophylactics, antidepressants, and serotonergic drugs, as well as sick leave and hospital admissions in non-users of triptans and users with and without triptan overuse.

## Methods

The study was approved by the ethics committee of the Medical University of Vienna. In order to analyse triptans and co-medications dispensed in 2007, we used the GAP-DRG database which includes data from 19 social security institutions in Austria. The analysis was based on prescriptions billed by pharmacies to the social security institutions. The GAP-DRG database includes all prescriptions covered by the insurances. For each package dispensed in 2007, the patients had to pay a prescription charge which was 4.70 Euros. The database does not include over-the-counter medication and prescription medication paid by the patients from their own pocket or dispensed free of charge to patients with severe chronic diseases or low income.

### Characteristics of subjects

For each insured person the following descriptors were available: anonymized unique identifiers of the patient receiving the drug and of the prescribing physician, the patients’ date of birth and sex, the postal code of her/his physical address, the Anatomical Therapeutic Chemical code (ATC) and the pharmacy article identifier of the dispensed drug (Pharmazentralnummer), the number of packages, details of sick leave (date and diagnosis) as well as the number of hospital admissions.

### Triptan use and overuse

We identified triptans and other medications by the ATC code and package size (units per package), dose per unit, and route of administration by the pharmacy article identifier. The first prescription of a triptan must be made by a neurologist; further prescriptions can be done by general practitioners and other physicians. In 2007, sumatriptan tablets, zolmitriptan tablets, melting tablets as well as nasal spray, eletriptan, and frovatriptan were available and reimbursed. Rizatriptan, naratriptan, sumatriptan injections, and sumatriptan suppositories were available but reimbursed in exceptional cases only. Sumatriptan nasal spray and almotriptan were not available.

We defined triptan use as ≥1 package of a triptan dispensed in 2007 and triptan overuse as ≥30 defined daily doses (DDD) dispensed in at least one quarter. According to the World Health Organisation (WHO) the DDD is defined as “the assumed average maintenance dose per day for a drug used for its main indication in adults” [21]. For acute medications the DDD is the average initial adult dose recommended [21]. Table 1 shows the specific DDD for each triptan according to these guidelines. The cut-off dose of 30 DDD was chosen in line with the International Classification of Headache Disorders (ICHD-3 beta) that requires the use of a triptan on 10 or more days per month over a period of three months for diagnosing triptan overuse headache [22]. As triptan use in the individual patient may be fluctuating over time, we assessed triptan overuse in one, two, three, and four quarters of 2007.

**Table 1:** Defined daily doses for each triptan available in Austria according to WHO guidelines [21]

	Formulation	Defined daily dose
Sumatriptan	50 mg tablet	1 tablet
	100 mg tablet	1 tablet
	10 mg nasal spray	not available
	20 mg nasal spray	not available
	25 mg suppository	1 suppository
	6 mg subcutaneous injection	1 injection
Zolmitriptan	2.5 mg tablet	1 tablet
	2.5 mg melting tablet	1 melting tablet
	5 mg nasal spray	1 spray
Eletriptan	20 mg tablet	2 tablets
	40 mg tablet	1 tablet
Frovatriptan	2.5 mg tablet	1 tablet
Rizatriptan	5 mg tablet	2 tablets^a^
	5 mg lyotablet	2 tablets^a^
	10 mg tablet	1 tablet
	10 mg lyotablet	1 tablet
Naratriptan	2.5 mg tablet	1 tablet

^a^ 1 tablet for patients taking propranolol therapy and for patients with mild to moderately decreased kidney or liver function

We compared the number of triptan users and overusers between the nine provinces of Austria as well as between predominantly urban, predominantly rural, and intermediate regions. These regions are categorised by the Statistik Austria based on the NUTS3 criteria (Nomenclature des unités territoriales statististiques) developed by the European Commission [23]. Furthermore, we assessed whether the prescribing physician was a general practitioner, neurologist, or other specialist.

### Use of other medications

We analysed the prescription of drugs used for migraine prophylaxis, antidepressants, and other serotonergic drugs in users and non-users of triptans. Migraine prophylactics comprised betablockers (propranolol, metoprolol, bisoprolol, atenolol), flunarizine, and antiepileptic drugs (topiramate, valproate). The inclusion of antidepressants had three reasons. First, some of them (e.g. amitriptyline and venlafaxine) are used as second-line migraine prophylactics. Second, comorbidity of migraine and depressive or anxiety disorders is common [2, 7, 9]. Third, the use of triptans together with serotonergic antidepressants may be associated with a serotonin syndrome [13]. For this reason, we included also other serotonergic drugs, namely fluanxol, ziprasidon, saquonavir, and lithium.

### General health status

To assess the general health status we compared the number of days of sick-leave and the number of hospital admissions in users and non-users of triptans comparing triptan overusers and non-overusers in the former group. The sick-leave diagnoses were made by general practitioners.

### Statistical analysis

Data was extracted from the GAP-DRG database and transferred into SPSS 24.0 (IBM SPSS Statistics, IBM Group 2016). We analysed data for all persons as well as for non-users and users of triptans separately, with the differentiation of triptan users into non-overusers and overusers. None of the continuous variables was normally distributed, therefore we used medians and quartiles (Q1, Q3) for descriptive analyses and Mann-Whitney-U tests for comparisons of different study groups. We calculated Chi^2^-tests for categorical variables and odds ratios (OR) for comparing the prescription of co-medication and hospital admissions in non-users of triptans and users without overuse (using non-users as reference) as well as in users without and with overuse (using users without overuse as reference).

## Results

### Triptan use and overuse

The database provided data of 7,426,412 million insured persons, covering 89.5% of all 8,295,189 inhabitants in Austria in 2007 [24]. The research population of persons aged 18–99 years with known sex and with insurance benefits in 2007 comprised 5,918,487 persons (90% of the inhabitants aged 18–99 years). Triptans were used by 33,062 persons (0.56% of the research population and 0.45% of all insured persons in the database) and overused by 1970 persons (i.e. 5.96% of all triptan users and 0.033% of the research population). Triptan users were significantly younger than non-users (median 44 vs. 47 years; *p* < 0.001) and they were more often female (82% vs. 54%; p < 0.001). Further details are given in Table 2. Comparisons between triptan users without and with overuse are given in Table 3. Triptan users without overuse refilled a median of 12 DDD per year, whereas triptan overusers refilled a median of 102 DDD per year (*p* < 0.001). Triptan overusers were significantly older than users without overuse (median 47 vs. 44 years; p < 0.001). Although triptans are not licensed for the use in persons older than 65 years we identified 1779 triptan users (5.4% of all users) and 164 overusers (8.3% of all overusers) in this age group. Triptan overuse in one, two, three or four quarters was found in 886 (45%), 409 (20.8%), 313 (15.9%), and 362 (18.4%) persons, whereas the corresponding median DDD (Q1; Q3) per year were 80 (64; 90), 108 (96; 117.5), 132 (120; 148.5), 174 (150; 220). The comparison of an overuse in one quarter to an overuse in two or more quarters showed an older age and – expectedly – a larger number of DDD in the latter (Table 4).

**Table 2:** Demographic data of triptan non-users and triptan users

	Triptan non-users *n* = 5,885,425		Triptan users *n* = 33,062		Statistics *p*-value
Age (years)					
Median	47		44		< 0.001^#^
Lower quartile	34		35		
Upper quartile	63		52		
Age group (years)	n	%	n	%	
18–35	1,619,098	27.5	8343	25.2	< 0.001^§^
36–50	1,698,601	28.9	15,134	45.8	
51–65	1,284,461	21.8	7806	23.6	
66–99	1,283,265	21.8	1779	5.4	
Sex					< 0.001^§^
Female	3,160,117	53.7	27,254	82.4	
Male	2,725,308	46.3	5808	17.6	

^#^Mann-Whitney-U-Test, ^§^Chi-Square Test

**Table 3:** Comparison of triptan users without and with overuse

	Triptan users without overuse *n* = 31,092		Triptan overusers *n* = 1970		Statistics *p*-value
Triptans DDD/year					
Median	12		102		< 0.001^#^
Lower quartile	6		84		
Upper quartile	32		132		
Range	1–106		30–762		
Age (years)					
Median	44		47		< 0.001^#^
Lower quartile	35		40		
Upper quartile	52		55		
Age group (years)	n	%	n	%	
18–35	8051	25.9	292	14.8	< 0.001^§^
36–50	14,216	45.7	918	46.6	
51–65	7210	23.2	396	30.3	
66–99	1615	5.2	164	8.3	
Sex					
Female	25,623	82.4	1631	82.8	0.666
Male	5469	17.6	339	17.2	

^#^Mann-Whitney-U-Test, ^§^Chi-Square Test, DDD/year defined daily doses per year

**Table 4:** Comparison of triptan overuse in one quarter and in two or more quarters of 2007

	Overuse in 1 quarter *n* = 886		Overuse in ≥2quarters *n* = 1084		Statistics p-value
Triptan DDD/year					
Median	80		132		< 0.001^#^
Lower quartile	64		110.3		
Upper quartile	90		162		
Range	30–132		60–762		
Age (years)					
Median	47		48		< 0.001^#^
Lower quartile	39		41		
Upper quartile	54		56		
Range	18–87		19–95		
Age group (years)	n	%	n	%	
18–35	153	17.3	139	12.8	0.002^§^
36–50	427	48.2	491	45.3	
51–65	245	27.7	351	32.4	
66–99	61	6.9	103	9.5	
Sex					0.761
Female	731	82.5	900	83	
Male	155	17.5	184	17	

^#^Mann-Whitney-U-Test, ^§^Chi-Square Test. *ns* not significant

### Regional differences

On the whole, the number of triptan users per 100,000 persons was 559 in Austria. In the nine provinces it ranged from 474 in Upper Austria to 640 in Vienna. The difference between the nine provinces was statistically significant (Chi^2^-test, *p* < 0.001). The proportion of triptan overusers was highest in Salzburg (10.7%) and lowest in Styria (3.0%). Triptan use was more common in urban than in rural and intermediate regions (Chi^2^-test, p < 0.001), whereas overuse did not differ between these regions (Chi^2^-test, *p* = 0.31); (Table 5).

**Table 5:** Research population and triptan use and overuse in the nine provinces and in rural, urban and intermediate regions

	Research population *n* = 5,918,487		Triptan users *n* = 33,062		Triptan overusers *n* = 1970		Users / 100,000 persons	Overusers among users
Province	n	%	n	%	n	%		%
Vienna	1,242,072	21.0	7956	24.1	348	17.7	640.5	4.4
Lower Austria	1,111,474	18.8	5722	17.3	560	28.4	514.8	9.8
Styria	874,749	14.8	4729	14.3	141	7.2	540.6	3.0
Upper Austria	922,842	15.6	4374	13.2	180	9.1	474.0	4.1
Tyrol	510,347	8.6	2989	9.0	140	7.1	585.7	4.7
Carinthia	406,493	6.9	2498	7.6	235	11.9	614.5	9.4
Salzburg	407,978	6.9	2363	7.1	253	12.8	579.2	10.7
Burgenland	194,083	3.3	1196	3.6	43	2.2	616.2	3.6
Vorarlberg	237,738	4.0	1201	3.6	68	3.5	505.2	5.7
Unknown	10,711	0.1	34	0.1	2	0.1	na	na
Region								
Rural	2,517,891	42.5	12,961	39.2	804	40.8	514.8	6.2
Urban	2,334,651	39.4	14,615	44.2	851	43.2	626.0	5.8
Intermediate	1,055,234	17.8	5452	16.5	313	15.9	516.7	5.7
Unknown	10,711	0.2	34	0.1	2	0.1	na	na

*na* not applicable

### Prescription patterns

Out of a total of 904,063 DDD of triptans dispensed in 2007, 95.7% were tablets, 1.3% were sumatriptan injections, 2.5% zolmitriptan nasal spray, and 0.4% sumatriptan suppositories; 675,146 DDDs (74.7%) were dispensed to non-overusers and 228,917 (25.3%) to overusers. Of 155,403 billed prescriptions, 91.4% were made for packages with six single doses, 41.2% for zolmitriptan, 32.5% for eletriptan, 14.8% for sumatriptan, 10% for frovatriptan, 1.2% for rizatriptan, and 0.4% for naratriptan.

In accordance with prescription rules, the majority of triptans was prescribed by general practitioners (113,649 prescriptions; 73.1%), followed by neurologists (23,139 prescriptions; 14.9%), and other specialists or hospital physicians (16,578 prescriptions; 10.7%). The prescriber could not be identified in 2037 (1.3%) of the prescriptions. In triptan overusers, 28,018 of 36,912 prescriptions (76.1%) were made by general practitioners, 5045 (13.7%) by neurologists and 3323 (9%) by other specialists.

### Co-medication

The drugs dispensed most often and second most often were betablockers and SSRI in non-users of triptans as well as SSRI and betablockers both in users without and with triptan overuse (Table 6). Any of the migraine prophylactics, antidepressants, and other serotonergic drugs specified in Table 6 were dispensed to 37% of the triptan users without overuse and to 54% of the overusers. All single drugs (Table 6) showed the same prescription pattern: Triptan users had a higher risk of being dispensed any of these co-medications than non-users, and triptan overusers had a higher risk than non-overusers. Looking at overusers more closely, 448 (50.6%) of those with an overuse in one quarter of 2007 and 611 (56.3%) of those with an overuse in ≥ two quarters had any of these co-medications (Chi^2^-test, *p* = 0.008).

**Table 6:** Co-medications dispensed to triptan non-users and to users without and with overuse

	Triptan non-users *n* = 5,885,425		Triptan users without overuse *n* = 31,092				Triptan users with overuse *n* = 1970			
	n	%	n	%	OR^#^	95% CI	n	%	OR^#^	95% CI
Betablockers	493,457	8.4	3455	11.1	1.4	1.3–1.4	376	19.1	1.9	1.7–2.1
Flunarizine	10,617	0.2	1425	4.6	26.6	25.1–28.6	162	8.2	1.9	1.6–2.2
Topiramate or valproate	26,605	0.4	1381	4.4	10.2	9.7–10.8	240	12.2	3.0	2.6–3.5
Tricyclic antidepressants	49,935	0.9	1319	4.2	5.2	4.9–5.5	166	8.4	2.1	1.8–2.5
SSRI	459,917	7.8	5596	18.0	2.6	2.5–2.7	444	22.5	1.3	1.2–1.5
SNRI	63,009	1.1	1412	4.5	4.4	4.2–4.6	116	5.9	1.3	1.1–1.6
NaSSA	85,402	1.5	1019	3.3	2.3	2.2–2.5	84	4.3	1.3	1.0–1.6
Other antidepressants or serotonergic drugs	168,020	2.9	2360	7.6	2.8	2.7–2.9	189	9.6	1.3	1.1–1.5

*OR*, odds ratio. *CI*, confidence interval. ^#^Odds ratios for triptan users without overuse refer to non-users (OR = 1) and odds ratios for triptan users with overuse refer to users without overuse (OR = 1) *SSRI*, selective serotonin re-uptake inhibitors, *SNRI,* serotonin-noradrenalin re-uptake inhibitors, *NaSSA*, noradrenergic and specific serotonergic antidepressants

### Sick leave

The number of days with sick leave due to any diagnosis as well as due to migraine (Table 7) was significantly greater in triptan users than in non-users (Mann-Whitney-U test, *p* < 0.001 for both). In contrast, days with sick leave in general and due to migraine did not differ between triptan users without overuse and triptan overusers. Similarly, the number of hospital admissions did not differ in the three subgroups (Table 7).

**Table 7:** Sick-leave and hospital admissions in triptan non-users and in users without and with overuse

	Triptan non-users n = 5,885,425			Triptan users without overuse n = 31,092			Triptan users with overuse n = 1970		
	Median	Q1	Q3	Median	Q1	Q3	Median	Q1	Q3
Sick-leave for									
any reason (days)	10	4	23	12	5	29	12	5	27.3
migraine (days)	2	1	4	3	1	7	4	1	9
	n	%		n	%	OR (CI)^a^	n	%	OR (CI)^#^
Hospital admissions	1,124,587	19.1		6082	19.6	1.0 (1.0–1.1)	386	19.6	1.0 (0.9–1.1)

Q1: 25% Quartile, Q3: 75% Quartile. *OR*, odds ratio. *CI*, confidence interval. ^a^Odds ratios for triptan users without overuse refer to non-users (OR = 1) and odds ratios for triptan users with overuse refer to users without overuse (OR = 1)

## Discussion

This is the first study that examined the prevalence of triptan use and overuse in the Austrian general population based on a nationwide healthcare database. Among people older than 18 years 0.56% were triptan users of whom nearly 6% were triptan overusers (0.033% of the general Austrian population). Triptan users were younger than non-users and they were more often female. Triptan overusers were older than non-overusers, whereas the female to male ratio was similar in the two groups. The prescription rates for triptans differed in the nine provinces of Austria and between urban, rural, and intermediate regions. The prescription of co-medications was lowest in triptan non-users, higher in users without overuse, and highest in triptan overusers. General health was poorer in triptan users and overusers than in non-users in terms of days of sick leave but not in terms of hospital admission rates.

### Triptan use

The rate of triptan use in Austria was lower than in other studies based on insurance claims. The rate of triptan overuse was comparable with other countries. A Dutch study found 1.3% triptan users and 0.1% overusers in the general population. The rate of triptan overusers among users was 10% according to IHS criteria and 3.3% according to more restrictive criteria requiring 18 DDD per month over a period of 3 months [14]. An Italian study found 0.7–1% triptan users of whom 10% – using at least 10 DDD per month – were classified as overusers [15]. Braunstein et al. [10] reported a rate of 2.3% triptan users in France; the proportion of overusers was 2.3% among all users and 5.4% among regular users. Differences between countries may be explained by methodological issues as well as by divergent insurance systems and prescription habits. In accordance with previous findings, the majority of triptan users was in the younger and middle age groups whilst triptan overusers were significantly older than non-overusers [10, 14, 15, 25]. Although triptans are not licensed in persons older than 65 years, 5.4% of the triptan users in our study were beyond this age. This finding is comparable with previous studies reporting triptan use in three to 10 % of people older than 65 years [10, 14, 15, 26]. In clinical practice, the use of triptans in this age group is not limited to otherwise healthy persons. Biagi et al. [27] found a substantial number of triptan users above 65 years with concomitant vascular risk factors. Even though the vascular risk of triptans is still under debate, triptans are contraindicated in vascular disorders [28, 29]. In addition, triptan overuse is not uncommon in persons older than 65 years. This age group accounted for 8.3% of all overusers in our study and for 12% of the overusers in the study from France [10].

The definition of triptan overuse varies in pharmacoepidemiological studies from ≥10, ≥ 15, ≥ 18, or ≥ 20 DDD per month to ≥30 DDD per quarter, or a conversion into ≥216 or ≥ 120 DDD per year, respectively [14–16]. We used quarterly DDD as a compromise between monthly DDD which may overestimate triptan overuse and annual DDD which may underestimate overuse if it is not present continuously [14, 15]. The fluctuation of triptan overuse is mirrored in our findings as well as in a previous study [15]. We found triptan overuse in one quarter of 2007 in 45% of the overusers and in two or more quarters in 55%. In the Dutch study, 63% to 65% of those who overused triptans in the first quarter of the year showed an overuse in at least one more quarter [14]. Defining triptan overuse by 30 or more DDD per quarter may still overestimate triptan overuse because patients may use more than one single dose per day. Therefore, the cut-off of 10 days of triptan use per month [22] may not be reached in each month. In our study. The lower quartile of DDD per quarter was 64 and the 5th percentile was 36. That suggests a low risk of overestimating triptan overuse in Austria.

The relation of 0.56% triptan users in our study to a migraine prevalence of 10% reported for Austria many years ago [30] suggests that less than 6% of the patients with migraine are using triptans in our country. This may reflect a low awareness of the options for treating acute migraine attacks among physicians and patients, and it may point out that some patients with migraine (but probably not 94%) respond well to analgesics and NSAIDS. A median of 12 DDD per year, i.e. one DDD per month, suggests a low to moderate use of triptans. A probable underuse of triptans in Austria is even more pronounced in men. The male to female ratio in the one-year-prevalence of migraine of 1:2.5 in Austria [30] contrasts with a ratio of 1:4.5 in the use of triptans in our analysis. This supports previous studies which showed that migraine is underdiagnosed and undertreated in men [31].

The low rate of triptan use contrasts with a considerable number of persons who overuse triptans. Triptan overusers accounted for 5.9% of all triptan users and for 25% of all dispensed triptans. This highlights the need of improving education about medication overuse headache for both patients and physicians.

### Regional differences

Our data cannot explain the differences in triptan use and overuse between the provinces as well as between urban and rural regions. In Burgenland and Carinthia, high rates of triptan users contrast with few available neurologists whereas in urban regions the higher rates of triptan users seem to be in line with a better availability of neurologists. For the treatment of migraine, patients may travel to another province or from rural to urban regions. In addition, socioeconomic differences not assessed in this study may play a role. Similar to our findings, DaCas et al. [15] showed a wide range of triptan use in different Italian regions without giving a specific explanation for the differences.

### Prescription patterns

The triptans prescribed most often in the present study were zolmitriptan and eletriptan. The same two were reported in France, whereas sumatriptan and rizatriptan ranked on top in the Netherlands as well as almotriptan and rizatriptan in Italy [10, 14, 15]. These prescription patterns may reflect differences in availability and reimbursement and suggest a common trend towards triptans with a faster onset of action [10, 14, 15]. In accordance with prescription rules and due to easier access, general practitioners prescribed the vast majority of triptans followed by neurologists. Compared to other countries, triptans were more often prescribed by neurologists [10, 14]. This may reflect an easier access to neurologists in Austria and may contribute to the lower rate of triptan overuse but also suggests that, compared to other countries, neurologists in Austria are more reluctant to prescribe triptans.

### Co-medication

Any of the migraine prophylactics, antidepressants, or other serotonergic drugs specified in Table 6 was dispensed to 37% of the triptan users without overuse and to 54% of the overusers. Hereby, physicians try to get in control of high- frequent headaches. However, patients with medication overuse headache have a higher prevalence of psychiatric comorbidity [6, 8, 25, 32]. Selective serotonin re-uptake inhibitors were most often dispensed presumably for depression or anxiety disorders. Among possible prophylactic drugs, betablockers were most often dispensed followed by flunarizine and tricyclic antidepressants. In triptan overusers, antiepileptic drugs came second. This supports findings of previous studies with prescriptions of antidepressants, prophylactics, and benzodiazepines in about one third of triptan users and even more often in triptan overusers [10, 14]. Although psychopharmacologic drugs may lead to a serotonin syndrome in combination with triptans [13], in everyday routine a substantial number of patients got triptans in combination with serotonergic drugs.

### Sick leave and hospital admissions

The number of days with sick leave for any reason as well as for migraine was higher in triptan users than in non-users. This supports the necessity of treating migraine and comorbid diseases adequately. The days with sick leave due to migraine should be interpreted with caution. Experience in daily practice suggests that patients may pretend other complaints due to the fear of losing their job if they miss work too often because of migraine. General practitioners are not allowed to share medical diagnoses with the employers but pressure amongst colleagues to tell about reasons for sick leave is high. Comparable to findings of Braunstein et al. [10], the days with sick leave did not differ between triptan users without and with overuse. The proportion of hospital admissions did not differ between triptan users and non-users although triptan users were significantly younger than non-users. This may indirectly point to a poorer health status in triptan users.

### Limitations of the study

Our study is limited by inhabitants not included in the research population, delays in billing dispensed drugs, the uncertainty if dispensed drugs were actually taken, and the fact that the database does not include diagnoses for outpatient treatments.

The findings in this study may have been biased because four small social security institutions are not included in the GAP-DRG database. These institutions, however, account for only 3% of all insured persons in Austria. The basis for our analysis comprised 89.5% of the general population in 2007. The study may have been biased by the fact that persons who were waived from prescription charges in 2007 could not be included. Usually, these are persons with lower socioeconomic status which was found to be associated with high frequency migraine, medication overuse headache, and psychiatric comorbidity [2, 26, 33].

Dispensed drugs were billed to the insurance by the pharmacies by the end of each quarter of the year. This may have led to missing data in one quarter and to carry-over effects in the next quarter. These deferrals are evened out over 12 months, but it may have influenced the DDD count for triptan overuse. Furthermore, we did not know, if the dispensed triptans were actually taken by the patients. Nevertheless, the finding that most patients refilled their prescription suggests that the dispensed triptans were taken.

Since the database does not include diagnoses for outpatient treatments or indications for medications, our inferences on migraine diagnoses were indirect. We may have misclassified cluster headache for migraine. Patients with cluster headache use triptans very frequently without being an overuser [34, 35]. The low prevalence of cluster headache and the finding that the triptans of choice for treating cluster headache, i.e. subcutaneous sumatriptan and zolmitriptan nasal spray, accounted for less than 4 % of the DDD suggest a low (if any) impact on our findings. In addition, we could not determine if persons fulfilling our definition of triptan overuse had medication overuse headache.

We analysed data from 2007 because at the time of the analysis this was the most recent complete data available. This data still reflects the current situation: In Austria no new formulations of triptans, other triptans, or other new groups of acute medications relevant for migraine have been marketed since 2007. In addition, a study by Fischer et al. found that in a tertiary care outpatient headache clinic in Innsbruck 73% of the migraineurs were triptan naive at their first consultation at the clinic in the years 2009 to 2012 [36].

### Strengths of the study

This is the first study in Austria and one of few nationwide studies in the literature looking at triptan use and overuse. In contrast to previous studies, the study covers the vast majority of the general population and is representative of the population structure as it includes nearly 90% of the inhabitants in the predefined age range of 18 to 99 years [10, 14–20]. In addition to the examination of triptan use and overuse, we were able to assess regional differences, prescription patterns of triptans, prescriptions of co-medications as well as data on sick leave and hospital admissions, and to compare these findings in non-users, users, and overusers of triptans.

## Conclusion

Overall triptan use and consequently overall triptan overuse was low in Austria but triptan users were at risk of overuse. Citing Leonard Cohen’s “Bird on the Wire”, some migraineurs may need the advice “Why not ask for more?” whereas others “You must not ask for so much”.

## Abbreviations

ATC code: Anatomical therapeutic chemical code
DALYs: Disability adjusted life years
DDD: Defined daily dose
GAP-DRG: General approach patient-oriented outpatient-based diagnosis related groups
ICHD-3: International Classification of Headache Disorders, 3rd version
NSAIDS: Non-steroidal antiiflammatory drugs
NUTS3: Nomenclature des unites territoriales statistiques
WHO: World Health Organisation
